# Supplementary material for: A mechanism-based computational model to capture the interconnections among epithelial-mesenchymal transition, cancer stem cells and Notch-Jagged signaling
Source: Oncotarget. 2018 Jul 6;9(52):29906–20. doi: 10.18632/oncotarget.25692 (PMC6057462; doi:10.18632/oncotarget.25692)
Supplement: Supplementary file 1 [file oncotarget-09-29906-s001.pdf]

# A mechanism-based computational model to capture the interconnections among epithelial-mesenchymal transition, cancer stem cells and Notch-Jagged signaling

## SUPPLEMENTARY MATERIALS

### Mathematical modeling of transcriptional/translational interactions

Transcriptional and translational activations/inhibitions in the circuit are modeled using shifted Hill functions introduced by Lu *et al* (1):

$$H^S(A,A_0,n,\lambda)=\frac{1}{1+\left(\frac{A}{A_0}\right)^n}+\lambda\frac{\left(\frac{A}{A_0}\right)^n}{1+\left(\frac{A}{A_0}\right)^n} \quad \text{(S1)}$$

In eq. S1, $A$ is the chemical species that poses the regulation and  is a half-maximal concentration. Further, the Hill coefficient $A_0$ models the smoothness of the interaction with respect to $A$. Lastly, $\lambda$ is the concentration fold change of the chemical species $X$ due to the interaction ($\lambda < 1$ for inhibition, $\lambda > 1$ for activation). To simplify the notation, only the first argument of the function $H^S$ is displayed in the main text.

### Mathematical modeling of post-translational interactions

Post-translational inhibitions happen when a micro-RNA species $\mu$ binds to a transcription factor or receptor/ligand. Such interactions are modeled via the function $P_l(\mu, n)$, where $n$ is the number of available binding sites for the micro-RNA $\mu$ on the inhibited chemical species. Additionally, the function $P_y(\mu, n)$ describes the corresponding decrease in the level of micro-RNA $\mu$ due to the degradation of the micro-RNA/mRNA complex. These functions are the standard modelling treatment of micro-RNA post-translational interactions in the MBC framework (microRNA-Based-Chimeric circuits) developed by Lu *et al* (1), and are defined as:

$$P_l(\mu,n)=\frac{L(\mu,n)}{Y_m(\mu,n)+k_m} \quad \text{(S2)}$$

$$P_y(\mu,n)=\frac{Y_\mu(\mu,n)}{Y_m(\mu,n)+k_m} \quad \text{(S3)}$$

where $L(\mu, n)$ is the total translation rate of the ligand/receptor, $Y_m(\mu, n)$ is the total active degradation rate of the messenger RNA of the ligand/receptor and $Y_\mu(\mu, n)$ is the total degradation rate of the micro-RNA:

$$L(\mu,n)=\sum_{i=0}^{n} l_i C_i^n M_i^n(\mu) \quad \text{(S4)}$$

$$Y_m(\mu,n)=\sum_{i=0}^{n} \gamma_{mi} C_i^n M_i^n(\mu) \quad \text{(S5)}$$

$$Y_\mu(\mu,n)=\sum_{i=0}^{n} \gamma_{\mu i} C_i^n M_i^n(\mu) \quad \text{(S6)}$$

In eqs. (S4–S6), $l_i$, $\gamma_{mi}$ and $\gamma_{\mu i}$ are individual rates for the case of micro-RNAs bound to the protein. Also, $C_i^n$ is the number of arrangements of $i$ molecules of micro-RNA in $n$ binding sites:

$$C_i^n=\frac{n!}{i!(n-i)!} \quad \text{(S7)}$$

and

$$M_i^n(\mu)=\frac{\left(\frac{\mu}{\mu_0}\right)^i}{\left(1+\frac{\mu}{\mu_0}\right)^n} \quad \text{(S8)}$$

where $\mu_0$ is a threshold for micro-RNA concentration. Please see the Supplementary Information of Lu *et al* (1) enabling specialized capabilities such as collective cell migration. Cell-fate determination between the three phenotypes is in fact regulated by a circuit composed of two highly interconnected chimeric modules-the miR-34/SNAIL and the miR-200/ZEB mutual-inhibition feedback circuits. Here, we used detailed modeling of microRNA-based regulation to study this core unit. More specifically, we investigated the functions of the two isolated modules and subsequently of the combined unit when the two modules are integrated into the full regulatory circuit. We found that miR-200/ZEB forms a tristable circuit that acts as a ternary switch, driven by miR-34/SNAIL, that is a monostable module that acts as a noise-buffering integrator of internal and external signals. We propose to associate the three stable states-(1,0 for a more thorough discussion of the MBC framework.

## REFERENCE

1. Lu M, Jolly MK, Levine H, Onuchic JN, Ben-Jacob E. MicroRNA-based regulation of epithelial-hybrid-mesenchymal fate determination. Proc Natl Acad Sci U S A. 2013; 110:18174–9.

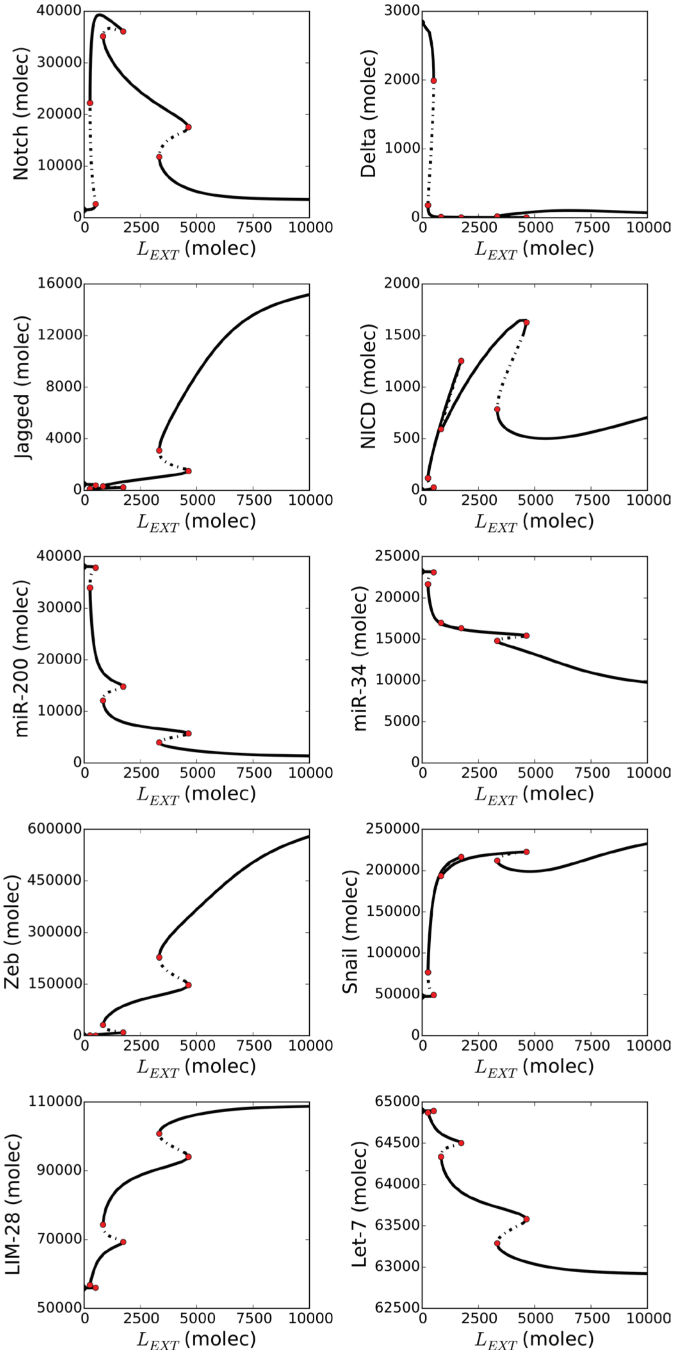


**Supplementary Figure 1: Bifurcation diagram of the model's variables vs external Notch ligands $L_{EXT}$ (Delta + Jagged).**

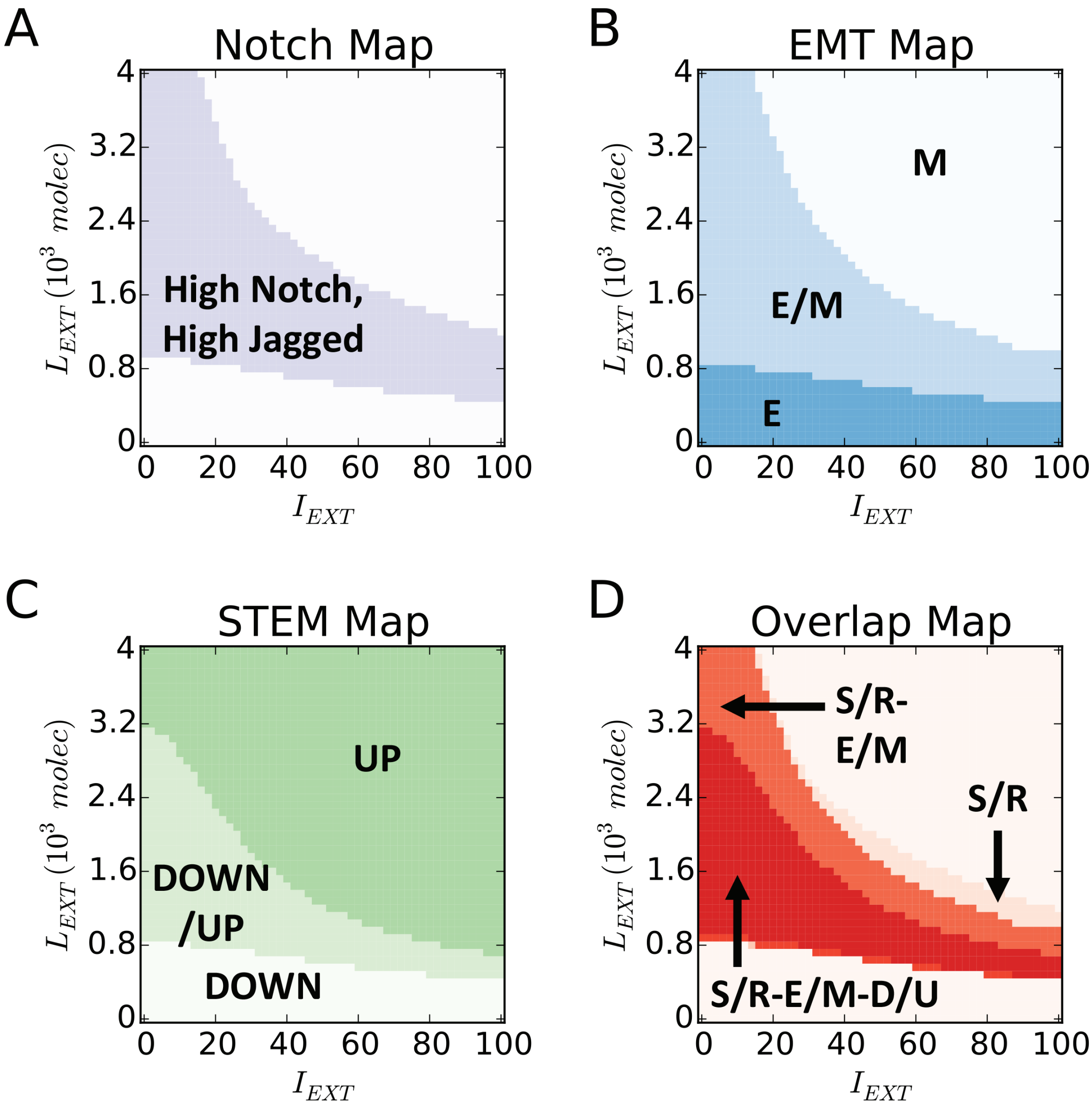


**Supplementary Figure 2:** (**A**) Phase diagram of the Notch state in presence of variable $I_{EXT}$ (x-axis) and Notch ligands $L_{EXT}$ (y-axis). Light red indicates the (high Notch, high Jagged) region. (**B**) Phase diagram of the EMT state. (**C**) Phase diagram of the Stem state. (**D**) Overlap of the three maps highlights the hybrid E/M-Stem-Sender/Receiver window. A large $I_{EXT}$ and/or overexpression of NF-κB pushes the cell out of the window in dark red. High Notch/high Jagged is abbreviated as S/R; STEM and UP are abbreviated as S and U. In this simulation, cell state is measured upon full equilibration. The initial condition is always E/M-D/U-S/R. NF-κb is fixed at the value 2.5 10i molecules that enable a coupled 'E/M-D/U-S/R' state at the reference point ($L_{EXT}$ = 2000 molecules, $I_{EXT}$ = 0 molecules).

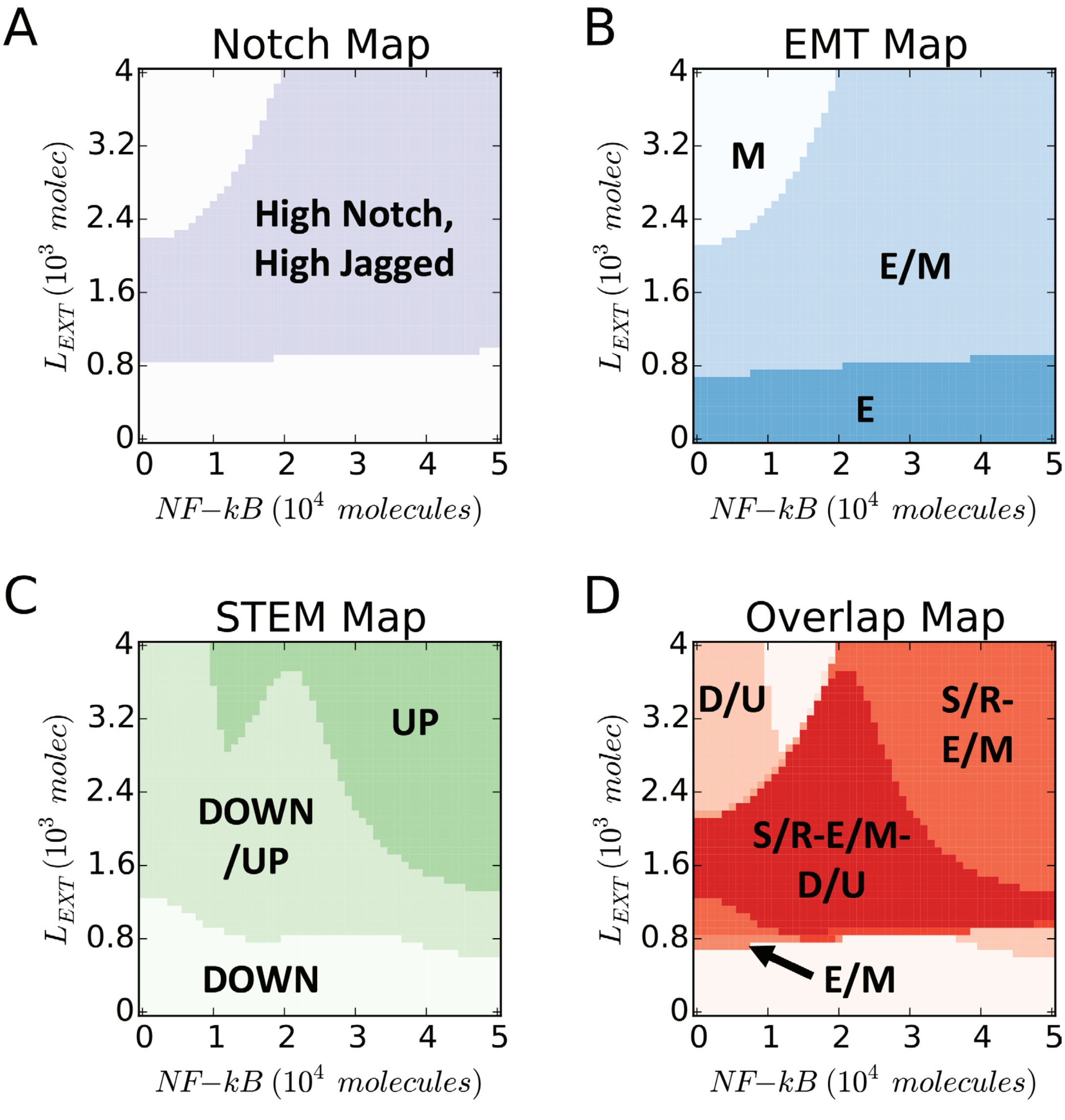


**Supplementary Figure 3:** (**A**) Phase diagram of the Notch state in presence of variable NF−kB (x-axis) and Notch ligands $L_{EXT}$ (y-axis). Light red indicates the (high Notch, high Jagged) region. (**B**) Phase diagram of the EMT state. (**C**) Phase diagram of the Stem state. (**D**) Overlap of the three maps highlights the hybrid E/M-Stem-Sender/Receiver window. A large $I_{EXT}$ and/or overexpression of NF-κB pushes the cell out of the window in dark red. High Notch/high Jagged is abbreviated as S/R; STEM and UP are abbreviated as S and U. In this simulation, cell state is measured upon full equilibration. The initial condition is always E/M-D/U-S/R. No EMT induction is applied ($I_{EXT}$ = 0 molecules) to enable a coupled 'E/M-D/U-S/R' state at the reference point ($L_{EXT}$ = 2000 molecules, NF-kB = 2.5 $10^4$ molecules).

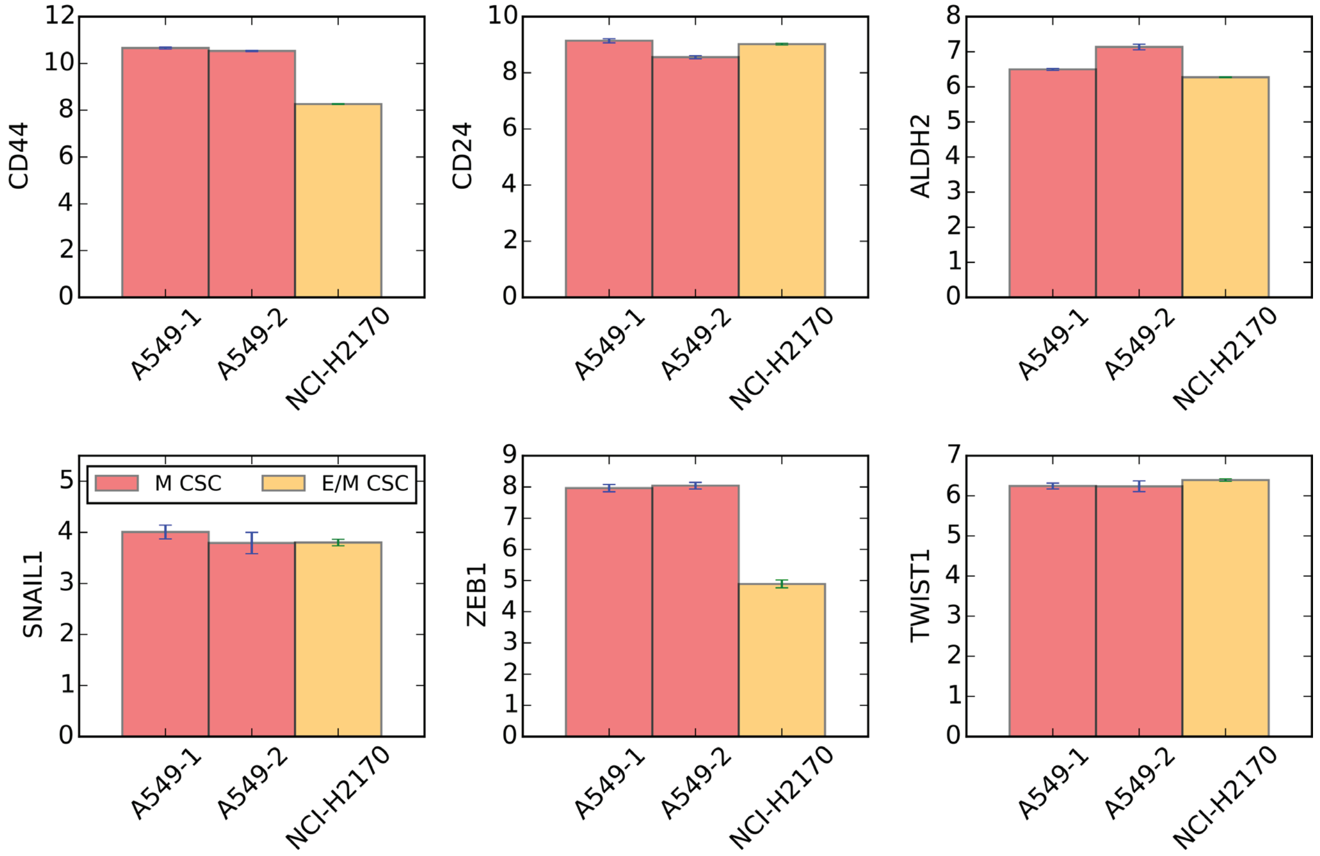


**Supplementary Figure 4: Expression of CD44, CD24, ALDH2, SNAIL1, ZEB1 and TWIST1 in the mesenchymal GSE50627 CSCs (in red, corresponding to A549 in Figure 5) and hybrid E/M GSE50627 CSCs (in yellow, corresponding to NCI-H2170 in Figure 5).** Error bars show the standard deviation among three technical replicates for each sample.

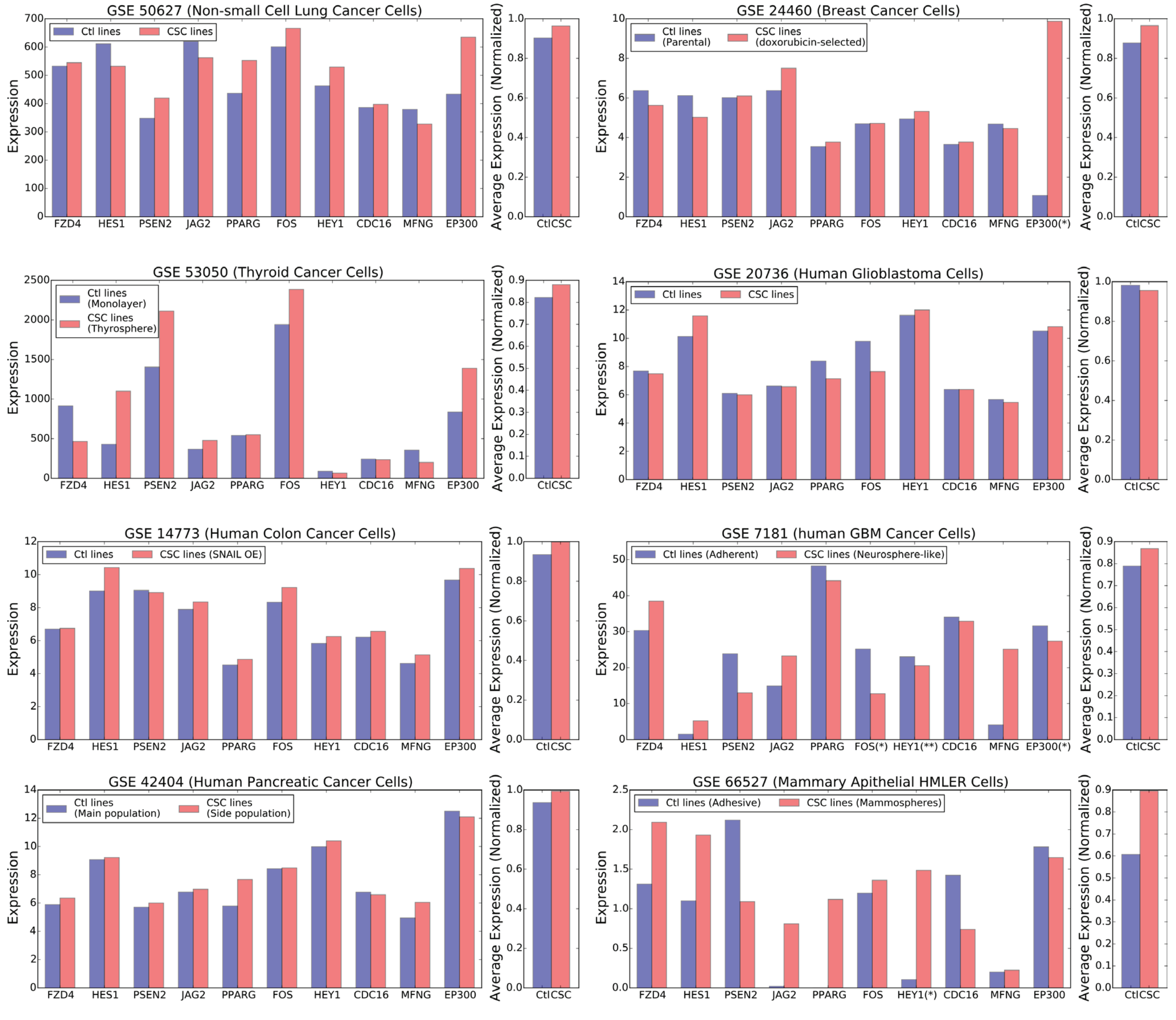


**Supplementary Figure 5: Left part of each panel: average expression of 10 genes that represent a signature for Notch signalling.** Blue bars represent the average in the control, non-stem cells (not plotted in Figure 5) while red represent the average in the CSCs considered in Figure 5. For each GSE dataset, the average was calculated over all the reported CSC subsets. Right part of each panel: overall average in the expression of the 10 genes in control, non-stem cells (blue) and CSCs (red). To compute the average, the levels of the 10 genes were normalized in [0, 1] first so that they all have the same weight.

**Table 1: Parameters of the Notch circuit**

| Parameter group | Parameter | Value | Dimensions |
|---|---|---|---|
| Degradation | $\gamma_N, \gamma_D, \gamma_J, \gamma_I, \gamma_U$ | 0.1, 0.1, 0.1, 0.5, 0.1 | $h^{-1}$ |
| Production | $g_N, g_D, g_J, U_0$ | 8, 20(#), 70(#), $10^5$ | $mRNAh^{-1}$ |
| Hill coefficient | $n_{I,N}, n_{I,D}, n_{I,J}, n_{I,U}, n_{U,N}$ | 2, 2, 5, 2, 2 | *Dimensionless* |
| Hill fold-change | $\lambda_{I,N}, \lambda_{I,D}, \lambda_{I,J}, \lambda_{I,U}, \lambda_{U,N}$ | 2, 0,2, 0.6, 0.8 | *Dimensionless* |
| Hill threshold | $I_{0,N}, I_{0,D}, I_{0,J}, I_{0,U}, U_{0,N}$ | 200, 200, 200, 200, $10^5$ | *Molecules* |
| Binding rates | $k_T, k_C$ | $110^{-4}$, $110^{-5}$ | $h^{-1}$ *Molecules*$^{-1}$ |
| Fringe | $n_F, \lambda_{F,D}, \lambda_{F,J}$ | 1.0, 3.0, 0.3 | *Dimensionless* |

**Table 2: Parameters of the EMT circuit**

| Parameter group | Parameter | Value | Dimensions |
|---|---|---|---|
| Degradation | $\gamma_S, \gamma_Z, \gamma_{\mu_{34}}, \gamma_{\mu_{200}}$ | 0.1, 0.1, 0.5, 0.5 | $h^{-1}$ |
| Production | $g_S, g, g_{\propto_{34}}, g_{\propto_{200}}$ | 90, 11, 1350, 2100 | $mRNAh^{-1}$ |
| Hill coefficient | $n_{Z,\propto_{34}}, n_{Z,\propto_{200}}, n_{S,\propto_{34}}, n_{S,\propto_{200}}, n_{Z,Z},$ $n_{S,S}, n_{S,Z}, n_{I,S}, n_{Iext,S}, n_{L7,Z}$ | 2, 3, 1, 2, 2, 1, 1, 2, 2 | *Dimensionless* |
| Hill fold-change | $\lambda_{Z,\mu_{34}}, \lambda_{Z,\mu_{200}}, \lambda_{S,\mu_{34}}, \lambda_{S,\mu_{200}}, \lambda_{Z,Z},$ $\lambda_{S,S}, \lambda_{S,Z}, \lambda_{I,S}, \lambda_{Iext,S}, \lambda_{L7,Z}(^*)$ | 0.2, 0.1, 0.1, 0.1, 7.5, 0.1, 10, 6.5, 6.5, 0.5(*) | *Dimensionless* |
| Hill threshold | $Z_{0,\propto_{34}}, Z_{0,\propto_{200}}, S_{0,\propto_{34}}, S_{0,\propto_{200}}, Z_{0,Z},$ $S_{0,S}, S_{0,Z}, I_{0,S}, Iext_{0,S}, I_{L7,Z}$ | 600*K*, 220*K*, 300*K*, 180*K*, 25*K*, 200*K*, 180*K*, 300, 300, 50*K* | *Molecules* (*K* = 1000) |

(*) indicates a parameter that was varied in the numerical solution of the model.

**Table 3: Parameters for translation, mRNA degradation and micro-RNA degradation upon protein-micro-RNA binding**

| Parameter group | Parameter | Value | Dimensions |
|---|---|---|---|
| Translation rate | $l_i$ | 1.0, 0.6, 0.3, 0.1, 0.05, 0.05, 0.05 | |
| mRNA degradation rate | $\gamma_{mi}$ | 0, 0.04, 0.2, 1.0, 1.0, 1.0, 1.0 | $h^{-1}$ |
| micro-RNA degradation rate | $\gamma_{\mu i}$ | 0, 0.005, 0.05, 0.5, 0.5, 0.5, 0.5 | $h^{-1}$ |

**Table 4: Parameters of the STEM circuit**

| Parameter group | Parameter | Value | Dimensions |
|---|---|---|---|
| Degradation | $\gamma_{L_{28}}, \gamma_{L_7}$ | 0.1, 0.05 | $h^{-1}$ |
| Production | $g_{L_{28}}, g_{L_7}$ | 20000, 200 | $mRNAh^{-1}$ |
| Hill coefficient | $n_{L_{28},L_{28}}, n_{L_7,L_7}, n_{L_{28},L_7}, n_{L_7,L_{28}}, n_{miR-200,L_{28}}, n_{NF-kB}$ | 7, 3, 2, 1, 2, 2 | *Dimensionless* |
| Hill fold-change | $\lambda_{L_{28},L_{28}}, \lambda_{L_7,L_7}, \lambda_{L_{28},L_7}, \lambda_{L_7,L_{28}}, \lambda_{miR-200,L_{28}}(^*), \lambda_{NF-kB}$ | 3, 11, 0.1, 0.1, 0.5($^*$), 2 | *Dimensionless* |
| Hill threshold | $I_{L_{28},L_{28}}, I_{L_7,L_7}, I_{L_{28},L_7}, I_{L_7,L_{28}}, I_{miR-200,L_{28}}, I_{NF-kB}$ | 200*K*, 12*K*, 500*K*, 25*K*, 10*K*, 25*K* | *Molecules* ($K$ = 1000) |

($^*$) indicates a parameter that was varied in the numerical solution of the model.

**Table 5: Parameters for Metformin inhibition on SNAIL and LIN-28**

| Parameter | Parameter | Value | Dimensions |
|---|---|---|---|
| Metformin Level | $M$ | 2 | *Dimensionless* |
| Hill Threshold | $M_0$ | 1 | *Dimensionless* |
| Hill coefficient | $n_M$ | 2 | *Dimensionless* |
| Hill fold-change | $\lambda_M$ | 0.5 | *Dimensionless* |

Since there is no feedback interaction to Metformin, the level and Hill threshold were taken as dimensionless since only their ratio matters (see definition of shifted Hill function).

## Characterization of Cancer Stem Cells in publicly available datasets

**Table 6: Characterization of stemness and corresponding reference for each dataset considered in Figure 5**

| Dataset | Characterization of stemness | Reference |
|---|---|---|
| GSE50627 | Markers CD166, CD44 and EpCAM | Zakaria N, Yusoff NM, Zakaria Z, Lim MN et al. *BMC Cancer* 2015 |
| GSE24460 | Markers CD44/CD24 | Calcagno AM, Salcido CD, Gillet JP, Wu CP et al. *J Natl Cancer Inst* 2010 |
| GSE53050 | Thyrosphere formation | Reeb NA, Lin RY Thyroid Disorders 2015 |
| GSE20736 | Culture of glioblastoma-initiating cells | Nogueira L, Ruiz-Ontañon P, Vazquez-Barquero A, Lafarga M et al. *Oncogene* 2011 |
| GSE14773 | Formation of spheres | Hwang WL, Yang MH, Tsai ML, Lan HY et al. *Gastroenterology* 2011 |
| GSE7181 | Formation of spheres | Beier D, Hau P, Proescholdt M, Lohmeier A et al. *Cancer Res* 2007 |
| GSE42404 | Markers CD45/CD31 | Van den Broeck A, Vankelecom H, Van Delm W, Gremeaux L et al. *PLoS One* 2013 |
| GSE66527 | Markers CD24/CD44 | Grosse-Wilde A, Fouquier d'Hérouël A, McIntosh E, Ertaylan G et al. *PLoS One* 2015 |
